# Noninvasive Intracranial Source Signal Localization and Decoding with High Spatiotemporal Resolution

**DOI:** 10.34133/cbsystems.0206

**Published:** 2025-04-09

**Authors:** Hao Zhang, Xue Wang, Guowei Chen, Yanqiu Zhang, Xiqi Jian, Feng He, Minpeng Xu, Dong Ming

**Affiliations:** ^1^ Academy of Medical Engineering and Translational Medicine, State Key Laboratory of Advanced Medical Materials and Devices, Tianjin International Joint Research Centre for Neural Engineering, and Tianjin Key Laboratory of Brain Science and Neural Engineering, Tianjin University, Tianjin, China.; ^2^ Haihe Laboratory of Brain-Computer Interaction and Human-Machine Integration, Tianjin, China.; ^3^ School of Biomedical Engineering and Technology, Tianjin Medical University, Tianjin, China.

## Abstract

High spatiotemporal resolution of noninvasive electroencephalography (EEG) signals is an important prerequisite for fine brain–computer manipulation. However, conventional scalp EEG has a low spatial resolution due to the volume conductor effect, making it difficult to accurately identify the intent of brain–computer manipulation. In recent years, transcranial focused ultrasound modulated EEG technology has increasingly become a research hotspot, which is expected to acquire noninvasive acoustoelectric coupling signals with a high spatial and temporal resolution. In view of this, this study established a transcranial focused ultrasound numerical simulation model and experimental platform based on a real brain model and a 128-array phased array, further constructed a 3-dimensional transcranial multisource dipole localization and decoding numerical simulation model and experimental platform based on the acoustic field platform, and developed a high-precision localization and decoding algorithm. The results show that the simulation-guided phased-array acoustic field experimental platform can achieve accurate focusing in both pure water and transcranial conditions within a safe threshold, with a modulation range of 10 mm, and the focal acoustic pressure can be enhanced by more than 200% compared with that of transducer self-focusing. In terms of dipole localization decoding results, the proposed algorithm in this study has a localization signal-to-noise ratio of 24.18 dB, which is 50.59% higher than that of the traditional algorithm, and the source signal decoding accuracy is greater than 0.85. This study provides a reliable experimental basis and technical support for high-spatiotemporal-resolution noninvasive EEG signal acquisition and precise brain–computer manipulation.

## Introduction

Electroencephalography (EEG) is a direct measurement of neuronal activity based on electrodynamics with a millisecond temporal resolution; however, scalp EEG has a low spatial resolution on the order of centimeters due to the presence of the volume conductor effect [1]. Specifically, the EEG signal obtained through the scalp is the sum of potentials discharged by tens of thousands of neurons within the brain, the main component of which is the postsynaptic potential of the neuron but also includes action potentials, posterior polarizations, and presynaptic potentials [2]. The exact distribution of the current sources within the cranium of a given scalp EEG signal cannot be reconstructed, which makes it difficult to achieve a finer level of brain–computer manipulation. To this end, researchers have developed a series of scalp EEG traceability algorithms for the localization of cerebral power signals, but because the number of acquisition electrodes is much smaller than the number of unknown intracranial sources, there is no unique solution to this inverse problem. Therefore, the unsuitable and nonunique solution of the EEG traceability problem directly affects the spatial resolution of scalp EEG and similarly constrains the accuracy of fine brain–computer manipulation intention recognition.

Transcranial focused ultrasound (tFUS) is a newly emerging neuromodulation technology that is capable of noninvasively delivering mechanical forces in the form of sound waves to specific regions of brain tissue to achieve modulation and treatment of intracranial target areas. With the advantages of high focusing precision, multiple focusing targets, and noninvasiveness, tFUS is able to achieve millimeter-level spatial resolution, intracranial multitarget focusing, and noninvasive neuromodulation and therapy [3]. The history of tFUS research can be traced back to the 1950s, with Lynn’s pioneering research laying the foundation for the subsequent development of ultrasound research in the field of neuroscience [4–6]. Based on the magnitude of ultrasound focal domain energy, tFUS can be classified into high-intensity focused ultrasound (HIFU) and low-intensity focused ultrasound (LIFU). HIFU produces irreversible coagulative necrosis of tissues at the focal site mainly through thermal effects and is generally used for ablative treatment of tumor tissues [7,8]. LIFU reversibly excites or inhibits neural activity in specific brain regions primarily through mechanical effects without damaging brain tissue. In this study, the intensity of the ultrasound used was much less than that of LIFU, which would not affect neuronal firing. Ultrasound modulated electroencephalography (USMEEG) is essentially an electrical signal that belongs to the category of neurophysiological signals, and our current study focuses only on electrophysiological signals and has not been carried out to investigate the effect of local cerebral blood flow. We will investigate whether there is a correlation between USMEEG and cerebral blood flow [9,10]. However, both HIFU and LIFU cause problems such as focus shift and lack of energy in the focal domain due to the strong acoustic attenuation and inhomogeneity of the cranium, so early tFUS studies removed some of the cranium in the path of the acoustic beam [11]. In recent years, with the development of magnetic resonance imaging, computed tomography, and multiarray phased-array technology, numerical simulation-guided noninvasive transcranial focusing with tFUS has gradually become possible [12,13].

**Fig. 1.**
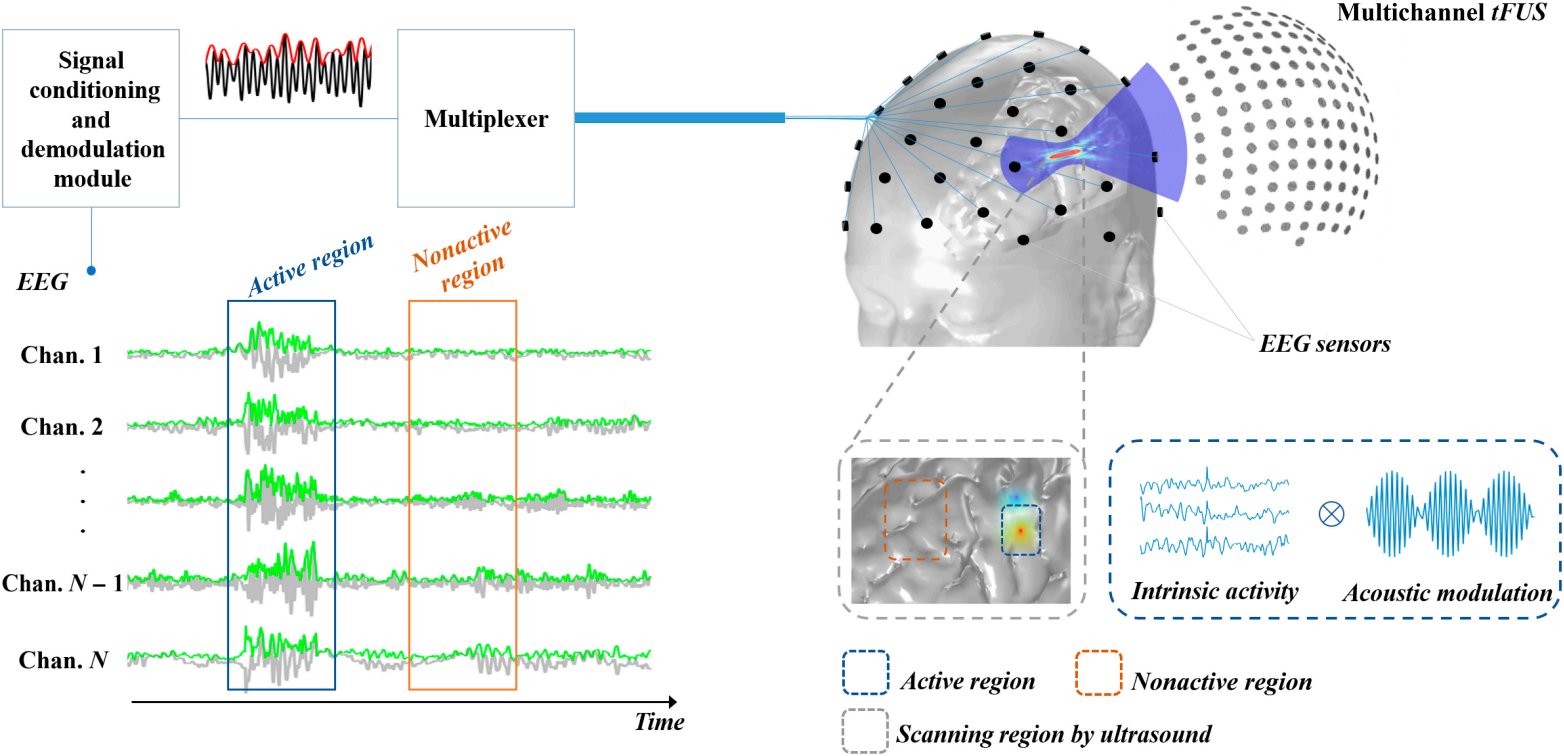
Schematic diagram of the ultrasound modulated electroencephalography (USMEEG) principle. EEG, electroencephalography; tFUS, transcranial focused ultrasound; Chan., channel.

tFUS is capable of focusing and modulating electrophysiological signals in the target area within the skull with a high spatial precision, thereby markedly improving the spatial resolution of EEG [14], and is a promising technique for acquiring high-spatial-and-temporal-resolution USMEEG signals; Fig. 1 shows a schematic of the principle of this technique. The acoustic field interacts with the electric field based on the acoustoelectric effect (AE) to produce ultrasound-encoded electrophysiological signals in the region of ultrasound irradiation. Ultrasound causes the presence of USMEEG encoding high-frequency ultrasound information in the activated regions of the scanned brain area by modulating the conductivity of the scanned brain area, whereas the output of the inactivated regions is noise. By decoding the acquired USMEEG, researchers are able to obtain the electrophysiological source distribution. Compared to traditional scalp EEG, USMEEG is able to selectively target electrophysiological sources to a particular brain region, improving the spatial resolution of scalp EEG without interfering with intracranial brain power signals.

In 2008, Olafsson et al. [15] firstly introduced the concept of ultrasound current source density imaging. In 2016, Qin et al. [16] demonstrated the feasibility of USMEEG for locating neuronal firing within the brain. In 2018, Burton et al. [17] achieved USMEEG data acquisition using both invasive and noninvasive electrodes, validating its capability to map hippocampal current sources in rats. In 2020, Zhou et al. [18] conducted saline experiments testing multisource dipoles of different frequencies and amplitudes, showing that USMEEG can localize dipoles of various amplitudes and frequencies and extract corresponding ultrasound features from the USMEEG signals, thus confirming the feasibility of multisource USMEEG. In 2021, Song et al. performed steady-state visual evoked potential experiments on live rats using USMEEG. Their results demonstrated high-amplitude responses at the fundamental and harmonic frequencies of visual stimuli in decoded USMEEG signals, marking the first instance of millimeter-scale-spatial-resolution steady-state visual evoked potential measurements in live rats using USMEEG [19]. In 2022, Zhang et al. [20] simulated brain tissue and neuronal discharges using tissue phantoms and platinum electrodes, achieving precise localization and decoding of single- and dual-source dipoles based on USMEEG with positioning errors less than 0.3 mm. In 2023, Zhang et al. employed a 128-element ultrasound phased-array system for transcranial ultrasound field modulation, successfully achieving transcranial localization of single- and dual-source dipoles in 2-dimensional space. Experimental results showed positioning errors less than 0.4 mm for single-source dipoles with a decoding accuracy exceeding 0.93 and less than 0.2 mm for dual-source dipoles with a decoding accuracy above 0.89, thereby demonstrating USMEEG as an effective tool for localizing regions of stimulated neuronal excitation [21].

In this paper, a numerical simulation and experimental study of USMEEG was carried out based on an ultrasonic phased array using tFUS modulated conventional EEG. Previous studies ignored the complex structure of a real skull, and the experimental scenarios mainly focused on 2-dimensional simulations and experiments, using traditional envelope algorithms in data processing with unstable localization and decoding effects. In contrast, this study innovatively explored the effects of a skull and simulated brain tissues on acoustoelectric signals based on a real skull structure, realized precise transcranial ultrasound focusing using phased-array ultrasound, and extended the simulation and experiments to 3 dimensions in order to be close to the actual scene. The pulse repetition frequency (PRF) features of acoustoelectric signals were further introduced, and a PRF sideband algorithm was developed to realize high-temporal-and-spatial-resolution noninvasive transcranial source signal localization and decoding. Specifically, a tFUS experimental platform was constructed based on a real brain model and a 128-array phased-array transducer in order to achieve precise control of focusing under both water-only and transcranial conditions. An experimental platform for USMEEG was also constructed, and a high-precision USMEEG dipole localization algorithm based on the sideband of the PRF was developed. In summary, this study solves the nonunique solution problem of the traditional EEG inverse problem from the source based on USMEEG and provides a theoretical basis and technical solution for further application to high-precision brain–computer manipulation by exploiting the high-resolution spatiotemporal information embedded in the source signal.

## Materials and Methods

### New York head model

**Fig. 2.**
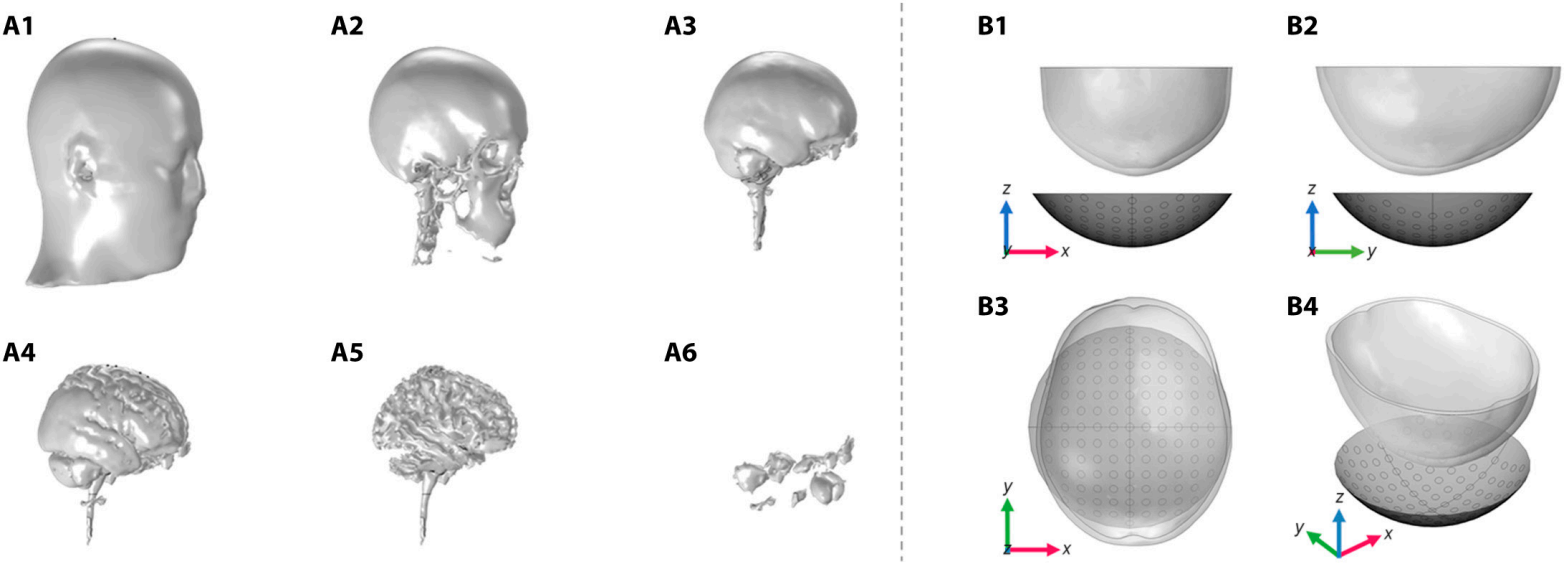
Schematic diagram of the structural forms of the New York head model and schematic diagram of the numerical simulation model. (A1) Scalp. (A2) Skull. (A3) Cerebrospinal fluid. (A4) Cerebral gray matter. (A5) Cerebral white matter. (A6) Cavities. (B1) *XZ* plane view. (B2) *YZ* plane view. (B3) *XY* plane view. (B4) Three-dimensional (3D) view.

The skull model used in this study was based on the New York head model. The New York head model is a finite element model developed based on the International Consortium for Brain Mapping (ICBM) 152 template developed by Huang et al.’s team [22] at the City University of New York. The researchers combined a nonlinear high-resolution brain image template [23] (ICBM152 v2009, 0.5-mm^3^ spatial resolution) and a high-resolution image template of noncerebral regions [24] (ICBM152 v6, 1-mm^3^ spatial resolution), which in turn was combined with the average head model of 26 subjects provided by Huang et al. [25] to extend the overall model size to the neck, resulting in the composite model ICBM-NY, the New York head model. The segmented structures of the New York head model include the scalp, skull, cerebrospinal fluid, cerebral gray matter, cerebral white matter, and cavities. Fig. 2A shows a schematic diagram of the morphology of each structure of the New York head model. In this study, we used the New York head model files in NIFTI format provided by the Parra Laboratory of the City University of New York; in order to increase the computational speed and save the computational resources, a portion of the cranium was intercepted for numerical simulation and subsequent 3-dimensional (3D) printing.

### Numerical simulation model of the sound field

Fig. 2B shows a schematic diagram of the numerical simulation model built based on the k-Wave simulation toolbox.

**Fig. 3.**
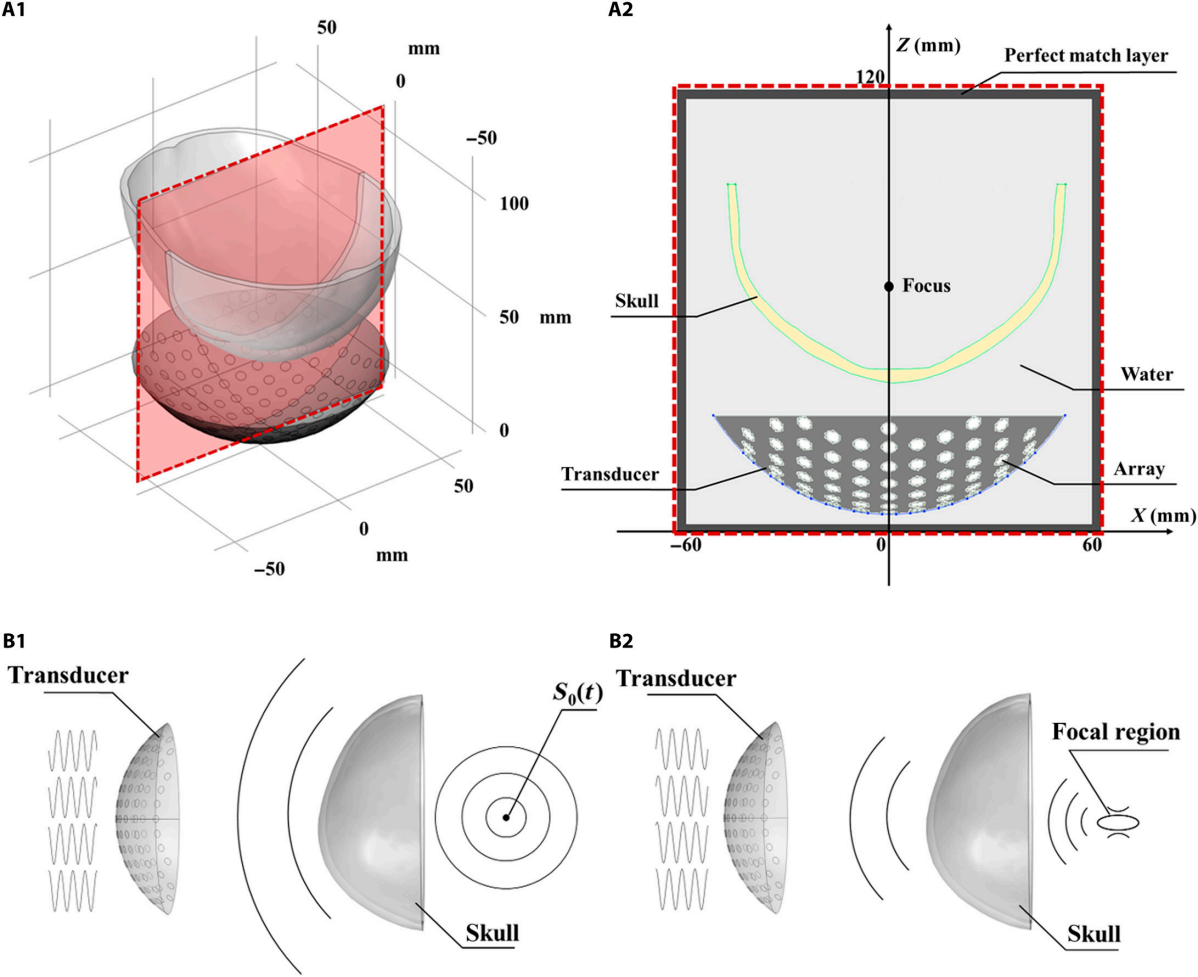
Schematic of the numerical simulation model for the *Y* = 0, *XZ* section and schematic diagram of the time reversal (TR) method. (A1) Three-dimensional view of the overall simulation model. (A2) *Y* = 0, *XZ* section details. (B1) Point source emission, received sound pressure at each array element. (B2) Focusing with TR.

Fig. 3A shows the numerical simulation model with *Y* = 0 and *XZ* cross-section. The overall opening diameter of the phased-array transducer is 100 mm, the radius of curvature is 60 mm, the number of array elements is 128, the center frequency is 1.1 MHz, the diameter of individual array elements is 4 mm, the center distance of the array elements is 8 mm, and the geometric focal point is set to be (0, 0, 60); the spatial step of the numerical simulation is 0.2 mm, and the time step is 10 ns. The specific simulation parameters are shown in Table 1.

**Table 1.** Numerical simulation parameters [35]

Material	Density (kg/m^3^)	Acoustic speed (m/s)	Acoustic impedance (MPa s/m)	Attenuation coefficient (Np/m/MHz)
Water	1,000	1,483	1.5	0.02
Skull	1,875	2,300	4.4	81

### Regulation methods

According to the Huygens–Fresnel principle, it is known that sound waves emitted by all array elements cause vibrations at a point in space by coherence and superposition at that point. This study is based on the time reversal (TR) method to adjust the phase of the excitation signals of each array element to correct the phase aberrations caused by the cranial bone, so as to achieve precise focusing of multichannel ultrasound. Fig. 3B shows a schematic diagram of the TR method.

In this study, a time series of sound pressure from a reflected source is received and the time series is inverted and re-emitted to achieve precise focusing of ultrasound. Firstly, a standard point sound source is placed at the preset focus as a function of

$$S_{0}\left(t\right)=I_{0}\text{sin}\omega t$$ (1)

where $I_{0}$ is the unmodulated initial intensity of each array element of the transducer and sinωt is the standard sinusoidal signal.

Each array element receives the sound pressure signal from the point source and fits the sound pressure signal received by the *i*th array element to $P_{i}\left(t\right)$, and then any one of the array elements is selected as the reference array element and set to $P_{\mathrm{ref}}\;\left(t\right)$. The $P_{\mathrm{ref}}\;\left(t\right)$ signal is autocorrelated, and the sound pressure fitted signals of the remaining array elements are cross-correlated with $P_{\mathrm{ref}}\;\left(t\right)$ by the following equation:

$$P_{\mathrm{ref}\cdot \mathrm{ref}}\left(t_{\mathrm{ref}}\right)=\int P_{\mathrm{ref}}\;\left(t\right)P_{\mathrm{ref}}\left(t-t_{\mathrm{ref}}\right)\mathrm{dt}$$ (2)

$$P_{\mathrm{ref}\cdot i}\left(t_{\mathrm{ref}\cdot i}\right)=\int P_{\mathrm{ref}}\;\left(t\right)P_{\mathrm{ref}}\left(t-t_{\mathrm{ref}\cdot i}\right)\mathrm{dt}$$ (3)

where $t_{\mathrm{ref}}$ is the autocorrelation signal delay of the reference array element and $t_{\mathrm{ref}\cdot i}$ is the intercorrelation signal delay of the remaining array elements with the reference array element. The excitation signal delay for each array element is obtained by calculating the difference between the intercorrelation signal delay of each array element and the autocorrelation signal delay of the reference array element:

$$\Delta t_{i}=t_{\mathrm{ref}\cdot i}-t_{\mathrm{ref}}$$ (4)

The modulated excitation signals of each array element are finally obtained:

$$S_{\mathrm{ip}}\left(t\right)=I_{0}\text{sin}\omega \left(t+\Delta t_{i}\right)$$ (5)

### Safety threshold

For the safety consideration of the tFUS modulation process, this study analyzed safety in terms of both the mechanical index (MI) and equivalent thermal dose (ETD) $t_{43}$. Among them, MI is a reference index used to reflect the mechanical effect of the ultrasound beam. When an ultrasound beam irradiates tissue, it is considered that the tissue does not show cavitation damage when the MI of the ultrasound beam is not greater than 1.9 [26]. MI is calculated by the formula

$$\mathrm{MI}=\frac{p_{-}}{\sqrt{f}}$$ (6)

where $p_{-}$ is the peak negative sound pressure and f is the center frequency of the ultrasonic transducer.

ETD is used to reflect temperature as a function of time and is used to evaluate the thermal damage index during ultrasound irradiation [27], and it is believed that brain tissue with an ETD of less than 10 does not show thermal damage [27]. ETD is calculated by the formula

$$t_{43}=\int_{0}^{t}R^{43-T_{t}}\mathrm{dt}$$ (7)

where $T_{t}$ is the temperature after ultrasonic irradiation, R=0.5 when $T_{t}>43\;{}^{\circ}C$, and R=0.25 when $T_{t}\leq 43\;{}^{\circ}C$ [28].

$$\rho c_{r}\frac{\partial T}{\partial t}=r\nabla^{2}T+Q+W_{B}c_{B}T$$ (8)

where r is the thermal conductivity, T is the temperature, $W_{B}$ is the perfusion rate of blood, and $c_{B}$ is the specific heat capacity of blood. Without considering blood perfusion, the Pennes equation can be simplified as

$$\frac{\partial T}{\partial t}=\frac{r}{\rho c_{r}}\nabla^{2}T+\frac{1}{\rho c_{r}}Q$$ (9)

where Q is the volumetric energy loss, which is calculated as

Q=2αI (10)

where α is the acoustic attenuation coefficient and the expression for I is given below:

$$I=\frac{1}{T_{p}}\int_{0}^{T_{p}}\frac{P^{2}}{\rho c}\mathrm{dt}$$ (11)

where $T_{p}$ is the period of the wave, P is the acoustic pressure, ρ is the density, and c is the acoustic speed.

### Sound field experimental platform

**Fig. 4.**
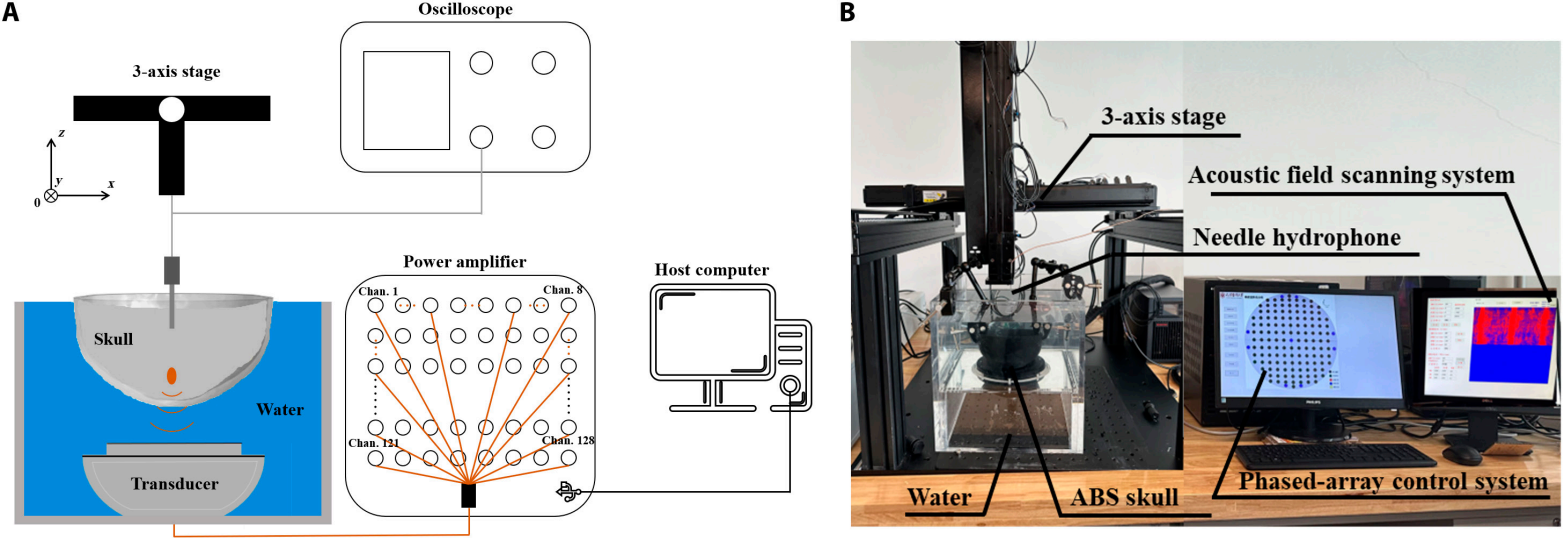
Sound field measurement system. (A) Schematic diagram of the sound field measurement platform. (B) Physical drawing of the sound field measurement platform. ABS, acrylonitrile butadiene styrene.

Fig. 4 shows a schematic diagram and a physical drawing of the acoustic field measurement platform; Fig. 4A shows a schematic diagram of the acoustic field measurement platform, and Fig. 4B shows a physical drawing of the acoustic field measurement platform. Among them, the phased-array transducer is a 128-array ultrasound phased array jointly developed by our laboratory and the Ultrasound Medicine Laboratory of Tianjin Medical University, with a diameter of 4 mm for a single array element, a focal length of 60 mm for the transducer, an opening diameter of 100 mm, and a center frequency of 1.1 MHz. The overall test system includes a phased-array control system, a hydrophone, a skull fixation device, an acrylonitrile butadiene styrene (ABS) imitation skull, 3D adjustment frame, a phased-array transducer, and an acoustic field scanning system. Among them, the ABS imitation skull was 3D printed from the skull model in the “Numerical simulation model of the sound field” section according to a ratio of 1:1, and the distance of the ABS imitation skull from the transducer was set to be 10 mm, and its center was aligned with the central arrays of the transducer along the direction of the acoustic axis. The acquisition range is the *XZ* section enclosed by the 4 points (−5, 0, 50), (5, 0, 50), (5, 0, 70), and (−5, 0, 70), where the scanning length in the *X* direction is 10 mm and that in the *Z* direction is 20 mm, with a scanning step of 0.2 mm. The acoustic field of the phased-array transducer in pure waters, the transcranial unmodulated acoustic field, and the transcranial acoustic field after phase modulation were detected under an initial excitation voltage of 0.5 V for each array element. Among them, the acoustic field in pure waters includes 3 central sections, *YZ*, *XZ*, and *XY*.

### The principle of ultrasound modulated EEG

Under the action of focused ultrasound (FUS) pressure, the conductivity of the irradiated region in the medium undergoes a periodic change, and the frequency of this periodic change depends on the parameters of ultrasound [29,30]. When FUS irradiates the medium, the conductivity of the medium in the focused region of FUS undergoes a periodic change, and the law of change is satisfied:

$$\frac{\Delta \rho }{\rho_{0}}=\mathrm{K\Delta P}$$ (12)

where $\rho_{0}$ is the initial conductivity of the medium, Δρ is the amount of change in the conductivity of the medium, ΔP is the change in the acoustoelectric pressure of the ultrasound, and K is the constant of the AE, which is usually 1 × 10^−9^ Pa^−1^ in NaCl solution. Therefore, when there is current flowing through the medium, the current in the irradiated region is modulated by the FUS, and the effect of the modulation is related to the sound pressure and frequency of the FUS, while the ultrasonic sound pressure in the region outside the focal domain is small, and the modulation effect is insignificant and negligible.

A pair of acquisition electrodes is referred to as a pair of leads, and the current density distribution between the leads can be called the lead field; according to the lead field theory, the voltage $V_{i}$ acquired by the *i*th acquisition electrode can be expressed as the following equation:

$$V_{i}=∭\rho \left({\tilde{J}}_{i}^{L}\cdot J^{I}\right)\mathrm{dx}\;\mathrm{dy}\;\mathrm{dz}$$ (13)

where $J^{I}$ is the current density distribution of the current source, $J^{I}=J^{I}\left(x,y,z\right)$; ${\tilde{J}}_{i}^{L}$ is the current density distribution of the conduction field, ${\tilde{J}}_{i}^{L}={\tilde{J}}_{i}^{L}\left(x,y,z\right)$; and ρ is the resistivity distribution, $\rho =\rho \left(x,y,z\right)$, which is given by Eq. (12):

$$\rho =\rho_{0}-\mathrm{K\Delta P}\rho_{0}$$ (14)

Substituting Eq. (14) into Eq. (13) gives

$$V_{i}=∭\rho_{0}\left({\tilde{J}}_{i}^{L}\cdot J^{I}\right)\mathrm{dx}\;\mathrm{dy}\;\mathrm{dz}+\;∭\left(-\mathrm{K\Delta P}\rho_{0}\right)\left({\tilde{J}}_{i}^{L}\cdot J^{I}\right)\mathrm{dx}\;\mathrm{dy}\;\mathrm{dz}$$ (15)

Eq. (15) can again be written as

$$V_{i}={V_{i}^{L}}^{F}+{V_{i}^{A}}^{E}$$ (16)

$${V_{i}^{L}}^{F}=∭\rho_{0}\left({\tilde{J}}_{i}^{L}\cdot J^{I}\right)\mathrm{dx}\;\mathrm{dy}\;\mathrm{dz}$$ (17)

$${V_{i}^{A}}^{E}=∭\left(-\mathrm{K\Delta P}\rho_{0}\right)\left({\tilde{J}}_{i}^{L}\cdot J^{I}\right)\mathrm{dx}\;\mathrm{dy}\;\mathrm{dz}$$ (18)

From Eqs. (16) to (18), it can be seen that the acquisition voltage $V_{i}$ consists of 2 components, ${V_{i}^{L}}^{F}$ and ${V_{i}^{A}}^{E}$, where ${V_{i}^{L}}^{F}$ is a low-frequency electrophysiological signal, which is generally an EEG component, and ${V_{i}^{A}}^{E}$ is a high-frequency USMEEG, whose signal frequency is in line with the ultrasound correlation frequency. Due to the large frequency difference between the 2 components, the 2 components can be separated by means of a filter. At this point, the high-frequency component of the measured voltage is obtained by high-pass filtering:

$${V_{i}^{A}}^{E}=-K∭\rho_{0}\left({\tilde{J}}_{i}^{L}\cdot J^{I}\right)b\left(x-x_{1},y-y_{1},z\right)P_{0}a\left(t-\frac{z}{c}\right)\mathrm{dx}\;\mathrm{dy}\;\mathrm{dz}$$ (19)

where $b\left(x,y,z\right)$ is the ultrasonic beam pattern, $P_{0}$ is the FUS acoustic pressure amplitude, $a\left(t\right)$ is the pulse waveform, and c is the medium acoustic speed.

Eq. (19) is the AE equation, which reflects the USMEEG as it relates to the ultrasound parameters and current source. The focal domain of FUS is approximated as an ellipsoid of millimeter size, and the acoustic pressure outside the focal domain is considered to be much smaller than the acoustic pressure inside the focal domain, so the effect of the acoustic pressure outside the focal domain on the conductivity of the medium is neglected. The integration over a 3D region is reduced to the computation of a 1-dimensional dot product by introducing a 3D Dirac function $\delta \left(x,y,z\right)$. Due to the screening properties of the Dirac function, the calculation of the magnitude of the USMEEG can be reduced to the calculation of the dot product of the current density distribution in the pairs of focal domains and the current density distribution in the conduction field. At this point, Eq. (19) can be written as

$${V_{i}^{A}}^{E}=K\rho_{0}\Delta P{\tilde{J}}_{i}^{L}\cdot J^{I}$$ (20)

When *N* lead fields exist, Eq. (20) can be written as

$$V_{\mathrm{AE}}=\left[V_{1}^{\mathrm{AE}},V_{2}^{\mathrm{AE}}\cdots V_{N}^{\mathrm{AE}}\right]$$ (21)

The conduction field current density distribution is

$$T=\left[{\tilde{J}}_{1}^{L},\;{\tilde{J}}_{2}^{L}\cdots {\tilde{J}}_{N}^{L}\right]$$ (22)

Substituting Eqs. (21) and (22) into Eq. (20) yields

$$V_{\mathrm{AE}}=K\rho_{0}\Delta \mathrm{PT}\cdot J^{I}$$ (23)

The current source density distribution can be obtained from Eq. (23) as

$$J^{I}=\frac{1}{K\rho_{0}\Delta P}T^{+}V_{\mathrm{AE}}$$ (24)

where $T^{+}$ is the Moore–Penrose pseudo-inverse matrix of T, which can be expressed as

$$T^{+}={\left(T^{T}T\right)}^{-1}T^{T}$$ (25)

The final current density distribution of the current source is obtained as the scatter of $J^{I}$:

$$\nabla \cdot J^{I}=-I$$ (26)

### PRF sideband localization decoding algorithm

**Fig. 5.**
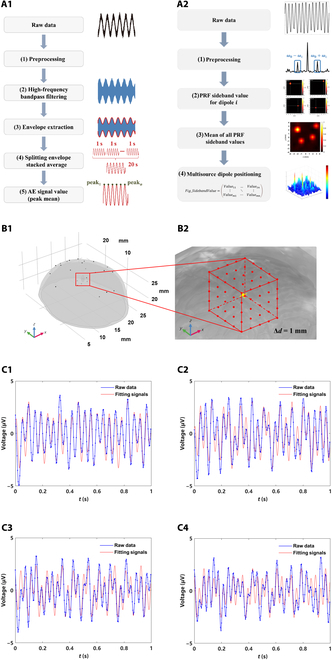
Schematic of the flow of the decoding and localization algorithm for acoustoelectric signals, 3D transcranial single-source dipole localization simulation model, and schematic diagram of the ultrasound irradiation strategy and electrocorticography (ECoG) signal compared to the fitted signal. (A1) Flowchart of the envelope decoding and localization algorithm [20]. (A2) Flowchart of the pulse repetition frequency (PRF) sideband localization algorithm. (B1) Numerical simulation model. (B2) Schematic diagram of the ultrasound irradiation strategy. (C1) S1 analog source signal. (C2) S2 analog source signal. (C3) S3 analog source signal. (C4) S4 analog source signal. AE, acoustoelectric effect.

The traditional USMEEG localization algorithm mainly uses the envelope information of the high-frequency acoustoelectric signal, and its main flow is shown in Fig. 5A1. From the acoustoelectric equation, the USMEEG is close to an amplitude-modulated (AM) signal. In this case, the USMEEG at the high frequency is the carrier, the source signal at the low frequency is the modulating signal, and the final signal acquired by the acquisition terminal is a bilateral-band AM signal. The frequency components of a double-sideband AM signal include the modulating signal component, the carrier component, the upper sideband, and the lower sideband. In the double-sideband AM signal obtained by acoustoelectric modulation, the carrier component is the PRF component, and the upper and lower sidebands are the 2 sidebands of the PRF.

The high-frequency carrier is assumed to be $m\left(t\right)$, and the low-frequency modulating signal is $\text{cos}\left(\omega_{c}*t\right)$, where $\omega_{c}$ is the source signal frequency.

$$m\left(t\right)=B_{0}*\text{cos}\left(\omega_{0}*t\right)$$ (27)

where $B_{0}$ is the ultrasound amplitude and $\omega_{0}$ is the PRF.

Given the dc component $A_{0}$, the bilateral-band AM time-domain and frequency-domain signals of the acoustoelectric modulation are obtained from Eq. (27), respectively:

$$S_{\mathrm{AM}}\left(t\right)=\left[A_{0}+m\left(t\right)\right]*\text{cos}\left(\omega_{c}*t\right)$$ (28)

$$\begin{array}{c} S_{\mathrm{AM}}\left(\omega \right)=\pi *A_{0}*\left[\delta \left(\omega +\omega_{c}\right)+\delta \left(\omega -\omega_{c}\right)\right]\\ +\frac{1}{2}*\left[M\left(\omega +\omega_{c}\right)+M\left(\omega -\omega_{c}\right)\right] \end{array}$$ (29)

where $M\left(\omega \right)=\pi *B_{0}*\left[\delta \left(\omega +\omega_{c}\right)+\delta \left(\omega -\omega_{c}\right)\right]$, which can be obtained by substituting the expression for $M\left(\omega \right)$ into Eq. (29):

$$\begin{array}{c} \begin{array}{c} \begin{array}{c} \begin{array}{c} S_{\mathrm{AM}}\left(\omega \right)=\pi *A_{0}*\left[\delta \left(\omega +\omega_{c}\right)+\delta \left(\omega -\omega_{c}\right)\right]+\frac{1}{2}*B_{0}*\delta \\ \left(\omega +\omega_{0}+\omega_{c}\right)+\delta \left(\omega -\omega_{0}+\omega_{c}\right)+\delta \left(\omega +\omega_{0}-\omega_{c}\right)+\delta \left(\omega -\omega_{0}-\omega_{c}\right) \end{array} \end{array} \end{array} \end{array}$$ (30)

From Eq. (30), when $\omega =\omega_{0}\pm \omega_{c}$, we know that

$$S_{\mathrm{AM}}\left(\omega \right)=\frac{1}{2}*B_{0}$$ (31)

A new USMEEG decoding and localization algorithm is designed based on the USMEEG bilateral sideband amplitude modulation model, which achieves accurate localization of dipoles based on the PRF sideband decoded values of USMEEG. When $\omega_{0}$ is given, for each source signal frequency $\omega_{c}$, the PRF sidebands have a specific frequency response, and this property markedly improves the signal-to-noise ratio (SNR) of each dipole source during multisource dipole localization, as shown in the flow in Fig. 5A2.

### USMEEG experimental platform

#### Numerical simulation model

The 3D transcranial single-source dipole numerical simulation model was constructed based on the above skull model and COMSOL 6.0 as shown in Fig. 5B1, and the ultrasound raster scanning area is shown in Fig. 5B2, with the yellow star shape as the neuron setting position and the red dots as the ultrasound irradiation point positions. Since the ultrasound focal domain is approximated as an ellipsoid, an ellipsoid-shaped region is used in the model to simulate the ultrasound focal domain, and the ellipsoid’s long and short axes are consistent with the measured acoustic field results, and ultrasound is used to perform 5 × 5 × 5 raster scanning with a scanning step of 1 mm. USMEEG was acquired by setting the domain point probes. where the electrical parameters of the scalp, skull, and brain tissues are shown in Table 2. The 4 dipole sources of S1, S2, S3, and S4 were set with the coordinates of (−1, −1, 0), (−1, 1, 0), (1, −1, 0), (1, −1, 0), and (1, 1, 0), respectively.

**Table 2.** Electrical parameters of the scalp, skull, and brain tissue

Electrical parameters	Scalp	Skull	Brain tissue
Conductivity (S/m)	0.59	0.27	0.78
Relative dielectric constant	36.43	19.06	38.26

#### Analog neuronal signal setup

To simulate neuronal discharges in the brain, discharge signals from neuronal nuclei in the skull were captured by electrocorticography (ECoG) as dipole discharge signals in the numerical simulation model [31]. The ECoG signal is fitted to the COMSOL analytical function as a dipole source discharge signal by MATLAB. The ECoG signal is plotted against the fitted signal as shown in Fig. 5C1 to C4, and the specific fitting equations obtained are as follows:

$$y^{j}=m^{j}+\sum_{i=1}^{k}\left(a_{i}^{\;j}*\text{cos}\left(\omega^{\;j}*t*i\right)+b_{i}^{\;j}\text{sin}\left(\omega^{j}*t*i\right)\right)$$ (32)

where k=8 is the fitting order and j is the dipole order number; the values of a and b are shown in Table 3, and the values of m and ω are shown in Table 4.

**Table 3.** Values of the fitted signal parameters a and b

Order	$a^{j=1}$	$b^{j=1}$	$a^{j=2}$	$b^{j=2}$	$a^{j=3}$	$b^{j=3}$	$a^{j=4}$	$b^{j=4}$
1	0.06526	0.12840	−0.1595	−0.0338	−0.2317	−0.1325	−0.2667	−0.3442
2	0.27110	−0.29910	−0.5398	−0.1985	−1.0085	−0.1612	0.4062	−0.5750
3	−0.12570	−0.22270	−0.9946	0.3627	0.0686	0.2248	0.2939	−0.0159
4	−0.33330	0.41430	0.2654	−0.0047	0.0193	0.1083	0.0116	−0.1595
5	−0.19810	−0.11340	−0.0491	0.1998	1.0539	1.0751	1.1008	0.7247
6	−0.07094	0.07147	0.1278	−0.1353	−0.0452	−0.0452	−0.0040	−0.0426
7	−0.05836	0.25900	0.8095	1.7175	0.0200	0.0190	−0.0197	0.0098
8	0.32070	2.01300	−0.0039	0.0059	0.0058	0.0264	0.0127	0.0035

**Table 4.** Values of fitted signal parameters m, *ω*, and goodness of fit

Source	m	ω	$R^{2}$
S1	3.5	17.25	0.89
S2	3.5	21	0.76
S3	3.5	32.8	0.71
S4	3.5	35.25	0.61

#### Experimental platform

**Fig. 6.**
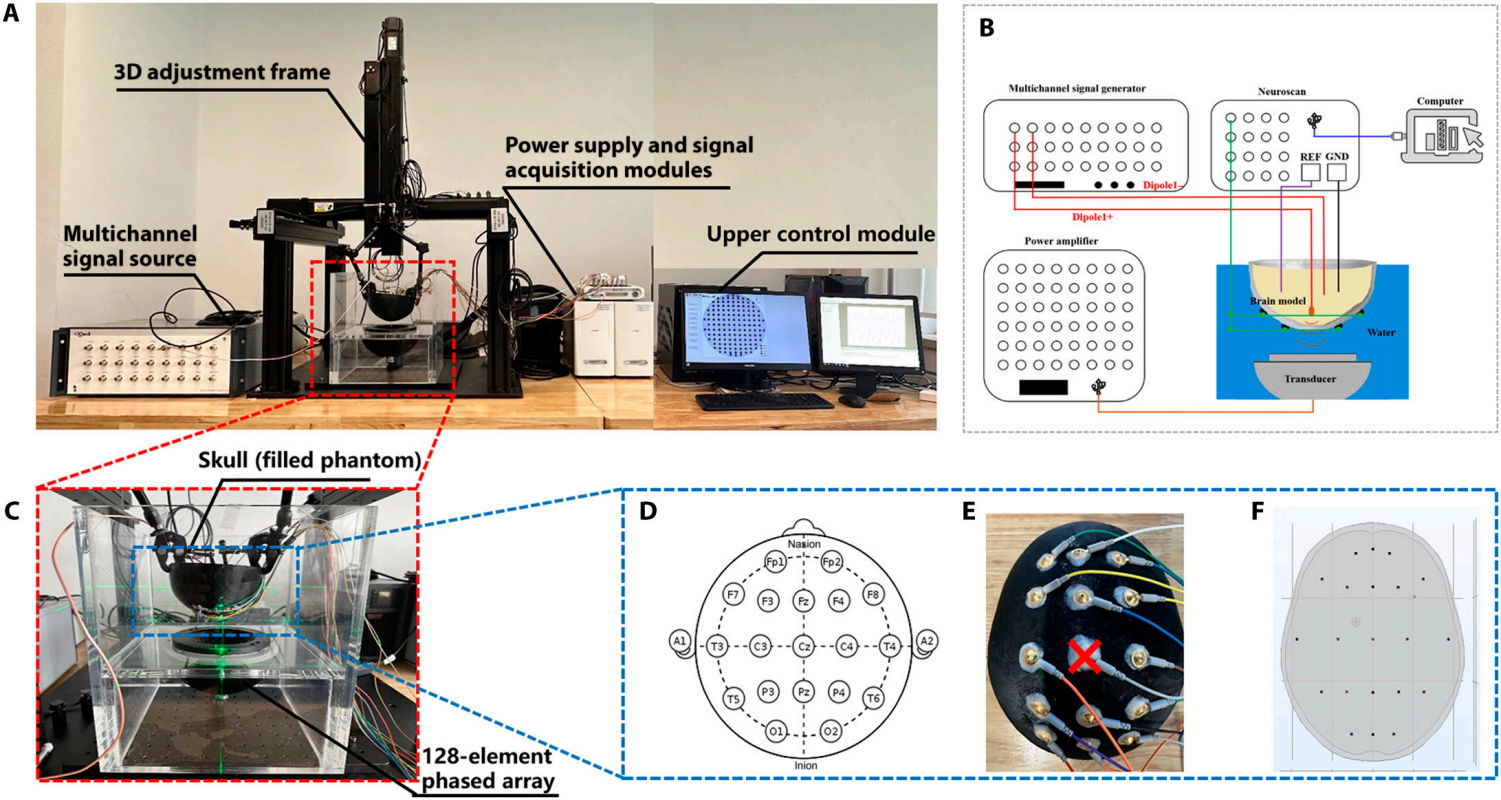
Schematic diagram of the ultrasonic phased-array experimental platform. (A) Physical drawing of the experimental platform. (B) Schematic diagram of the experimental platform connection. (C) Enlarged view of the skull fixation device. (D) Schematic diagram of the 10–20 international standard lead system. (E) Experimental lead distribution diagram. (F) Numerical simulation of lead distribution.

A schematic diagram of the experimental platform is shown in Fig. 6. Among them, Fig. 6A shows the physical diagram of the experimental platform, including a multichannel signal generator (QDAC-II, Qdevil, Denmark), a 3D adjustment frame, a signal acquisition module (Neuroscan Curry 9, USA), an upper computer control module, an ABS imitation skull (with internal filling of the body mold), a 128-element phased array, and an acrylic transparent water tank. The upper control module includes the phased-array control system, the acoustic field scanning system, and the SynAmps2 system. Fig. 6B shows the connection schematic of the experimental platform; Fig. 6C shows an enlarged view of the skull fixation device, which was used to ensure its stability; the experiments were performed using multiple-lead acquisition of USMEEG, and the lead placement potentials were referenced to the 10–20 international standard lead system, and Fig. 6D shows a schematic diagram of the 10–20 international standard lead system [32]; Fig. 6E shows the distribution of experimental leads, and Fig. 6F shows the distribution of numerical simulation leads. The experiments were performed by fixing the acquisition electrodes to the ABS imitation skull surface through Ten20 Fixed Conductive Paste, which reduces the impedance and fixes the electrode wires at the same time. A total of 14 leads were used to acquire USMEEG for the experiments, and the CZ lead was excluded because it was located in the ultrasound incidence path; a total of 21 leads were used to acquire USMEEG for the numerical simulations.

#### Experimental methods

Firstly, the input commands were compiled through the host computer, the multichannel signal generator was driven through the serial port, and the multichannel signal generator was used to connect the bayonet nut connector cable and the platinum wire electrode to output the multichannel sinusoidal signals as the multidipole source, and the platinum wire electrode was inserted into the specified position of the body mold, and the ultrasound was incident along the *Z* axis. The TR method was used to modulate the phased-array transducer to focus under transcranial conditions, and ultrasound was used to perform 5 × 5 × 5 raster scanning with a scanning step of 1 mm. The parameters of the multidipole setup at the time of the experiment are shown in Table 5.

**Table 5.** Table of experimental parameters for multisource dipole sources

Experimental parameters	S1	S2	S3	S4
Coordinates (mm)	(−1, 1, 0)	(1, 1, 0)	(1, −1, 0)	(−1, −1, 0)
Frequency (Hz)	8	11	13	15
Amplitude (mV)	500	500	500	500
Phase (°)	0	0	0	0

#### Data processing

The voltage signals acquired by Neuroscan at each point of the ultrasound scanning grid were firstly used as raw data; the raw data were preprocessed by downsampling at 4 kHz and trapping at 50 Hz. Fast Fourier transform was performed on the preprocessed signal to obtain its spectrum, the expected frequency range of the source signal was set from 0 to $\omega_{c}$, and the spectral value of the signal in the frequency band from PRF − $\omega_{c}$ to PRF + $\omega_{c}$ (with a frequency resolution of 1 Hz) was used as the USMEEG sideband decoding value under the frequency of the corresponding decoded source signal at the current point. The localization results were obtained by sideband decoding to each decoded source signal frequency and finally averaged and superimposed to obtain the dipole localization results in the 0 to $\omega_{c}$ band.

## Results

### Acoustic pressure field

**Fig. 7.**
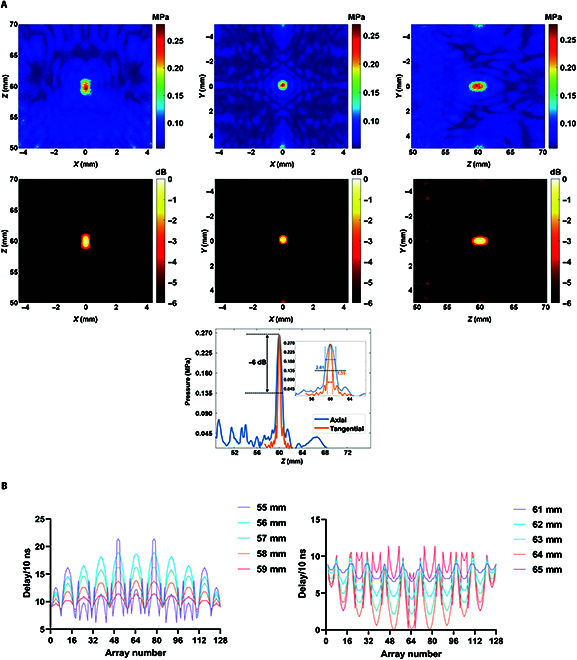
(A) Phased-array transducer pure water acoustic field. (B) Distribution of excitation delay curves for each array element of the ultrasonic phased array.

The phased-array transducer pure water acoustic field measurements are shown in Fig. 7A. From Fig. 7A, it can be seen that the focal domain distribution of the phased-array transducer approximates an ellipsoid, and the acoustic pressure extension *Z* axis shows a trend of increasing and then decreasing; among them, the focal point acoustic pressure is 0.27 MPa, and the long and short axes of the acoustic pressure −6 dB are 2.61 and 1.31 mm, respectively.

**Fig. 8.**
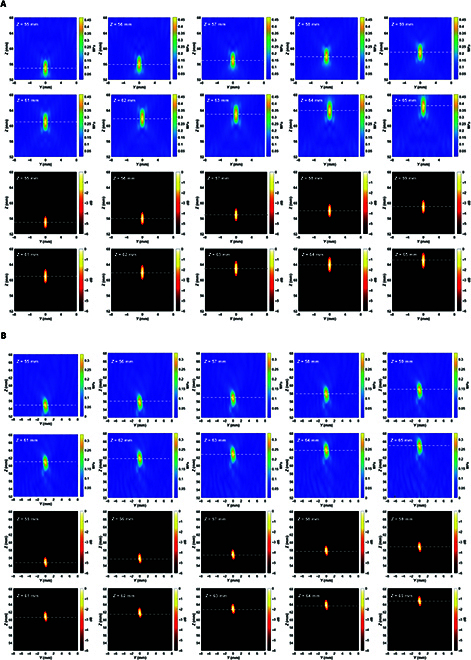
(A) Numerical simulation of focal modulation acoustic field distribution results. (B) Experimental focus modulation acoustic field distribution results.

The TR method was used to achieve regulation of the pure watershed focus to achieve focus of the tFUS to move on the *Z* axis, and the regulation ranges were set to be 55 to 59 and 61 to 65 mm; i.e., the focus coordinates were (0, 0, 55), (0, 0, 56), (0, 0, 57), (0, 0, 58), (0, 0, 59), (0, 0, 61), (0, 0, 62), (0, 0, 63), (0, 0, 64), and (0, 0, 65). The excitation delay for each regulation state is calculated by the k-Wave numerical simulation model as shown in Fig. 7B. The focusing results after numerical simulation modulation obtained by the above excitation delay are shown in Fig. 8A. Using the excitation delay parameters calculated by numerical simulation to drive each array element of the transducer, the results of the acoustic field after modulation in the experimental state are obtained as shown in Fig. 8B.

**Fig. 9.**
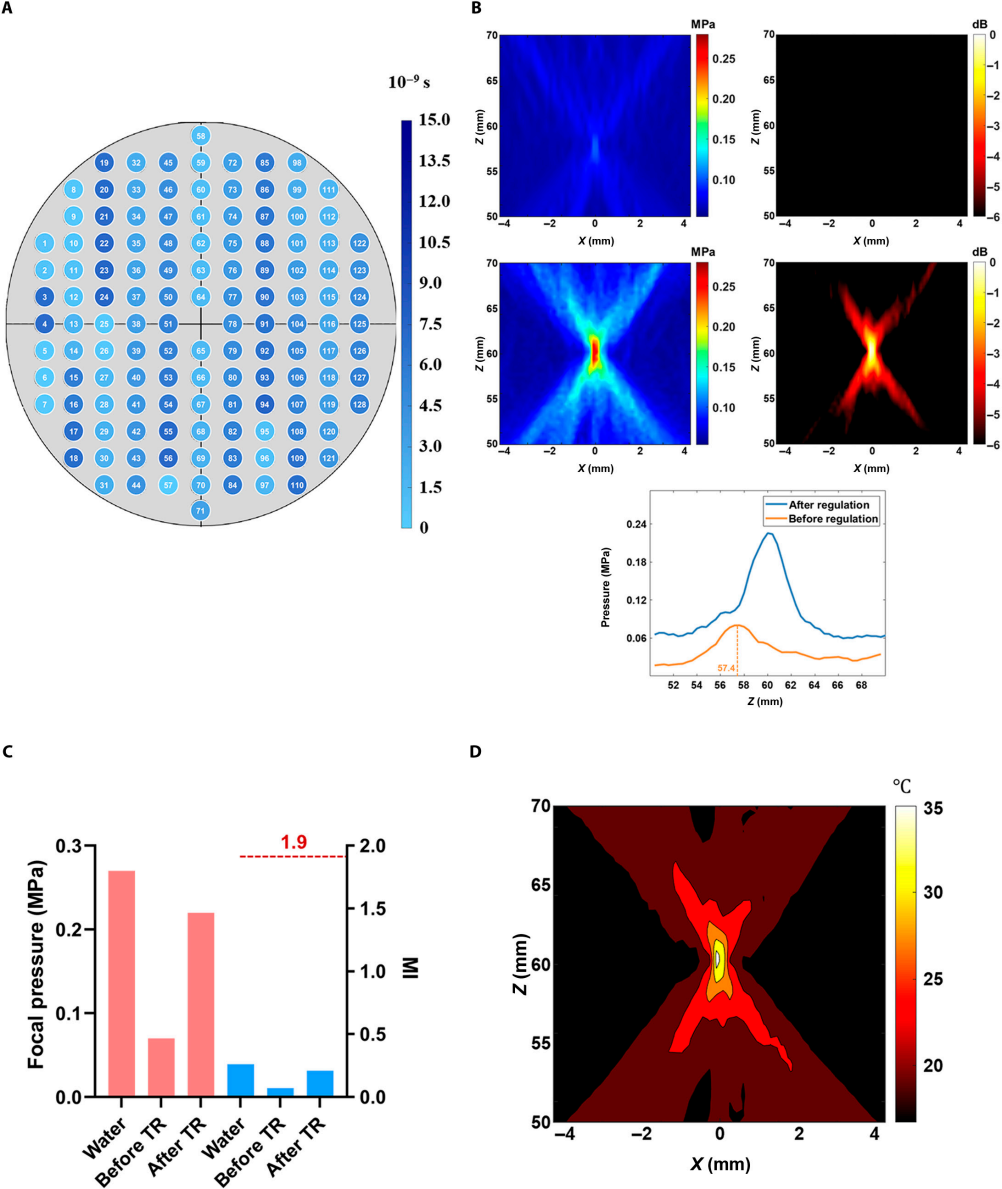
(A) Schematic of the delay distribution of the array elements (the darker the color, the higher the delay). (B) Acoustic pressure field before and after transcranial modulation. (C) Comparison results of the focal acoustic pressure and mechanical index (MI). (D) Distribution of transcranial TR-modulated onset temperature field.

Through the k-Wave simulation toolbox to set the point acoustic source transmit ultrasound, the acoustic pressure signal is received by each array element, and the excitation delay of each array element is obtained after the TR method is regulated, and the delay schematic of the array element is shown in Fig. 9A, in which each circular position is the real distribution position of the array element, and the serial number in the circle is the number of the array elements. Transcranial focusing is achieved by driving each array to emit ultrasound using excitation signals with different delays. The acoustic pressure field results before and after transcranial modulation are shown in Fig. 9B.

### Safety threshold

#### Mechanical index

The peak negative acoustic pressures of the phased-array transducer were measured to be 0.27, 0.07, and 0.22 MPa in pure waters, in the cranially unmodulated state, and after phased modulation, respectively, under the condition that the initial excitation voltage of each array element was 0.5 V. The peak negative acoustic pressures of the phased-array transducer were measured to be 0.27, 0.07, and 0.22 MPa. The MI calculated from Eq. (6) were 0.26, 0.07, and 0.21 for pure waters, transcranial unregulated state, and after phasic regulation, respectively, none of which was greater than 1.9 and all of which were within the safety threshold. The focal acoustic pressures and MIs for the 3 cases are shown in Table 6, and the comparison results are shown in Fig. 9C.

**Table 6.** Focal acoustic pressure and MI for each modulation method

Evaluation indicators	Pure water	Transcranial unmodulated	Transcranial TR modulation
Focal acoustic pressure (MPa)	0.27	0.07	0.22
MI	0.26	0.07	0.21

#### Equivalent thermal dose

Firstly, the starting temperature field corresponding to the stable acoustic field was calculated based on the Pennes heat transfer equation, and the temperature field was obtained as shown in Fig. 9D, and the focal point temperature at the current moment was calculated to be 20.6 °C. Based on this temperature, the ETDs of ultrasound continuous irradiation for 1 to 60 s were calculated as shown in Table 7. As shown in Table 7, within 60 s of ultrasound irradiation, the ETD orders of magnitude ranged from 10^−5^ to 10^−4^, none of which was greater than 10, which is within the safe threshold range.

**Table 7.** ETD statistics for ultrasound continuous irradiation 1 to 60 s

Span (s)	ETD	Span (s)	ETD	Span (s)	ETD
1	1.16 × 10^−5^	21	24.36 × 10^−5^	41	47.56 × 10^−5^
2	2.32 × 10^−5^	22	25.52 × 10^−5^	42	48.72 × 10^−5^
3	3.48 × 10^−5^	23	26.68 × 10^−5^	43	49.88 × 10^−5^
4	4.64 × 10^−5^	24	27.84 × 10^−5^	44	51.04 × 10^−5^
5	5.80 × 10^−5^	25	29.00 × 10^−5^	45	52.20 × 10^−5^
6	6.96 × 10^−5^	26	30.16 × 10^−5^	46	53.36 × 10^−5^
7	8.12 × 10^−5^	27	31.32 × 10^−5^	47	54.52 × 10^−5^
8	9.28 × 10^−5^	28	32.48 × 10^−5^	48	55.68 × 10^−5^
9	10.44 × 10^−5^	29	33.64 × 10^−5^	49	56.84 × 10^−5^
10	11.60 × 10^−5^	30	34.80 × 10^−5^	50	58.00 × 10^−5^
11	12.76 × 10^−5^	31	35.96 × 10^−5^	51	59.16 × 10^−5^
12	13.92 × 10^−5^	32	37.12 × 10^−5^	52	60.32 × 10^−5^
13	15.08 × 10^−5^	33	38.28 × 10^−5^	53	61.48 × 10^−5^
14	16.24 × 10^−5^	34	39.44 × 10^−5^	54	62.64 × 10^−5^
15	17.40 × 10^−5^	35	40.60 × 10^−5^	55	63.80 × 10^−5^
16	18.56 × 10^−5^	36	41.76 × 10^−5^	56	64.96 × 10^−5^
17	19.72 × 10^−5^	37	42.92 × 10^−5^	57	66.12 × 10^−5^
18	20.88 × 10^−5^	38	44.08 × 10^−5^	58	67.28 × 10^−5^
19	22.04 × 10^−5^	39	45.24 × 10^−5^	59	68.44 × 10^−5^
20	23.20 × 10^−5^	40	46.40 × 10^−5^	60	69.60 × 10^−5^

ETD, equivalent thermal dose

### Localization

**Fig. 10.**
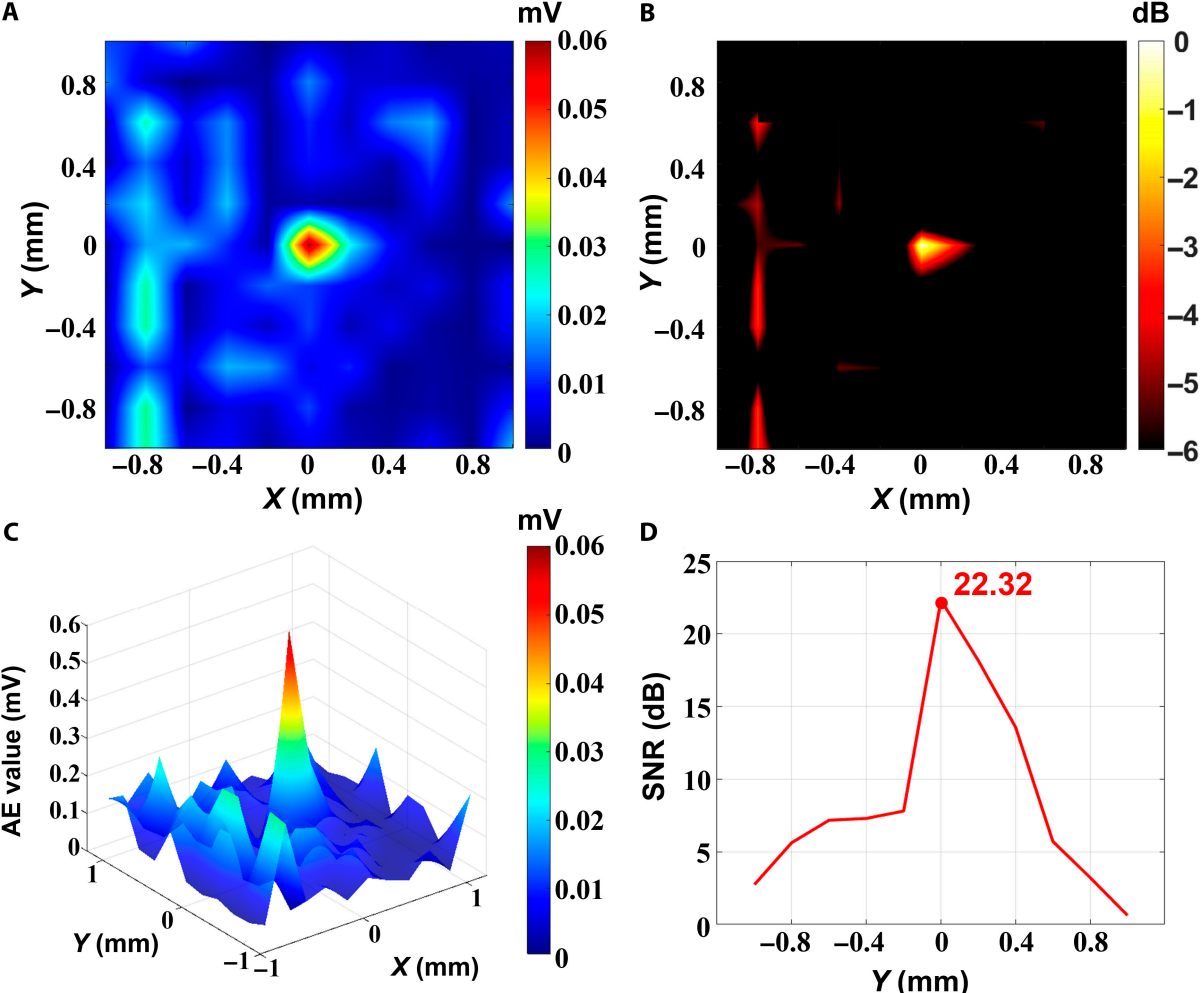
(A) Step 0.2 mm decoding USMEEG amplitude matrix. (B) Step 0.2 mm decoding USMEEG amplitude −6-dB decay heat map. (C) Three-dimensional surface distribution of decoded USMEEG amplitudes with a 0.2-mm step. (D) SNR distribution curve of decoded scanning points along the *Y* axis at *X* = 0.

Before the start of the formal experiment, we conducted a set of single-source dipole localization experiments based on a simulated brain tissue phantom to verify the effectiveness of the system. A 13-Hz single-source dipole was set in the center of the phantom, and ultrasound was performed with an 11 × 11 raster scanning, with a scanning step of 0.2 mm, and the localization results of the single-source dipole obtained by using the traditional USMEEG decoding method are shown in Fig. 10, where Fig. 10A shows the decoded USMEEG amplitude matrix with a step of 0.2 mm and it can be seen that the (0, 0) position has the largest value of 0.06 mV, which is the position of the dipole obtained by localization and is consistent with the electrode discharge position of the experimental setup; Fig. 10B shows the decoded USMEEG amplitude −6-dB decay thermogram with a step of 0.2 mm, and a clear target can be observed in the (0, 0) position; Fig. 10C shows the 3D surface distribution graph of the decoded USMEEG amplitudes with a step of 0.2 mm, which can more intuitively observe the location of the maximum value of the decoded USMEEG value; and Fig. 10D shows the distribution curve of the decoded SNRs of the scanning points distributed along the *Y* axis when *X* = 0, in which the position of (0, 0) has the largest SNR, which is 22.32 dB. This result verifies the effectiveness of the system.

#### Simulation

**Fig. 11.**
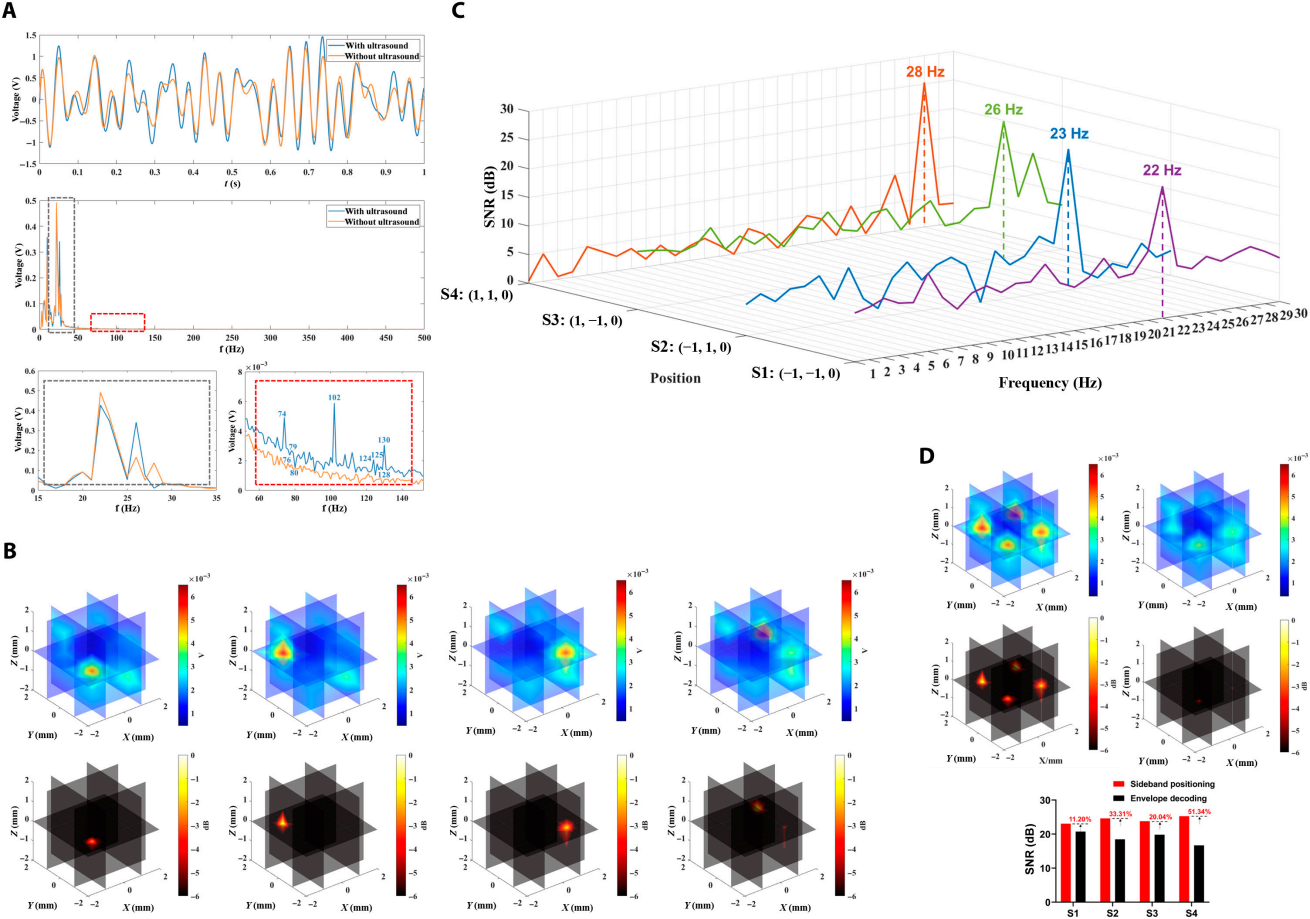
(A) Time–frequency plot of raw voltage signal with/without ultrasound irradiation. (B) Dipole localization results for 4 sources corresponding to sideband bands. (C) SNR curves for each sideband frequency at the set position of the source signal. (D) Comparison of PRF sideband localization and envelope decoding localization results.

The multisource dipole signals with a discharge frequency of 22 Hz and a set position of (−1, −1, 0), a discharge frequency of 23 Hz and a set position of (−1, 1, 0), a discharge frequency of 26 Hz and a set position of (1, −1, 0), and a discharge frequency of 28 Hz and a set position of (1, 1, 0) are collected as shown in Fig. 11A. From Fig. 11A, it can be seen that the voltage signal acquired without ultrasound irradiation does not have a band-specific signal component in the high-frequency band, and when there is ultrasound irradiation, under the influence of ultrasound modulation, the acquired voltage signal has a high-frequency component in the specific frequency band and has a specific frequency of ultrasound of PRF = 102 Hz, with the simultaneous presence of the spectral peaks of PRF ± 22, PRF ± 23, PRF ± 26, and PRF ± 28; i.e., 80 and 124, 79 and 125, 76 and 128, and 74 and 130 Hz, in total 8 sideband spectral peaks. The simulated USMEEG of each ultrasound scanning grid point (5 × 5 × 5, total 125 scanning points) was processed using the PRF sideband localization algorithm described above, and the expected source signal frequency range of 1 to 30 Hz was set to obtain the dipole localization results for each sideband band, where the dipole localization results for the 4 sources corresponding to the sideband bands are shown in Fig. 11B. As can be seen from Fig. 11B, the sideband acoustoelectric value localization results and −6-dB attenuation thermograms for the sideband frequencies PRF ± 22, PRF ± 23, PRF ± 26, and PRF ± 28 have clear target points at the set position where the corresponding source signals are located at (−1, −1, 0), (−1, 1, 0), (1, −1, 0), and (1, 1, 0), whereas the other sideband frequency localization results do not have clear targets, indicating that the PRF sideband acoustoelectric values are able to accurately localize the dipole positions at the corresponding frequencies. The calculated SNRs of the PRF sideband acoustoelectric values for each sideband frequency at each source signal set position are shown in Fig. 11C. Among them, the localization SNR of 22 Hz at (−1, −1, 0) is 23.05 dB, which is markedly higher than those of the other sideband frequencies; the localization SNR of 23 Hz at (−1, 1, 0) is 24.61 dB, which is markedly higher than those of the other sideband frequencies; the localization SNR of 26 Hz at (1, −1, 0) is 23.78 dB, which is markedly higher than those of the other sideband frequencies; and the localization SNR of 28 Hz at (1, 1, 0) is 25.26 dB, which is markedly higher than those of the other sideband frequencies.

The PRF sideband localization results are obtained by averaging and superimposing the frequency values of each sideband and comparing them with the dipole localization results obtained using the envelope decoding localization algorithm described above, which are shown in Fig. 11D. The specific values of the PRF sideband localization and envelope decoding localization for the 4 dipole sources localization SNRs are shown in Table 8.

**Table 8.** Table of SNR values for dipole source localization

Source	PRF sideband positioning	Envelope decoding for localization
S1-SNR/dB	23.05	20.73
S2-SNR/dB	24.61	18.46
S3-SNR/dB	23.78	19.81
S4-SNR/dB	25.26	16.69

From the above results, it can be seen that the −6-dB decay heat map of PRF sideband localization has clear targets at the dipole source setting positions, and it is considered that PRF sideband localization can accurately locate 4 dipole sources, while the −6-dB decay heat map of envelope decoding localization has clear targets only at the S1 and S3 setting positions, and it is considered that envelope decoding localization can accurately locate only 2 dipole sources, and the SNR of the other dipole sources is too low. Comparing the localization SNRs of the 4 dipole sources, for S1, the envelope decoding localization is 20.73 dB and the PRF sideband localization is 23.05 dB, which is an improvement of 11.20%; for S2, the envelope decoding localization is 18.46 dB and the PRF sideband localization is 24.61 dB, which is an improvement of 33.31%; for S3, the envelope decoding localization is 19.81 dB and for the PRF for S3, the envelope decoding is positioned at 19.81 dB and the PRF sideband is positioned at 23.78 dB, an improvement of 20.04%; and for S4, the envelope decoding is positioned at 16.69 dB and the PRF sideband is positioned at 25.26 dB, an improvement of 51.34%. Comparing the SNRs of the 4 dipole sources, the PRF sideband localization is markedly improved over the envelope decoding localization in all cases.

#### Experiment

**Fig. 12.**
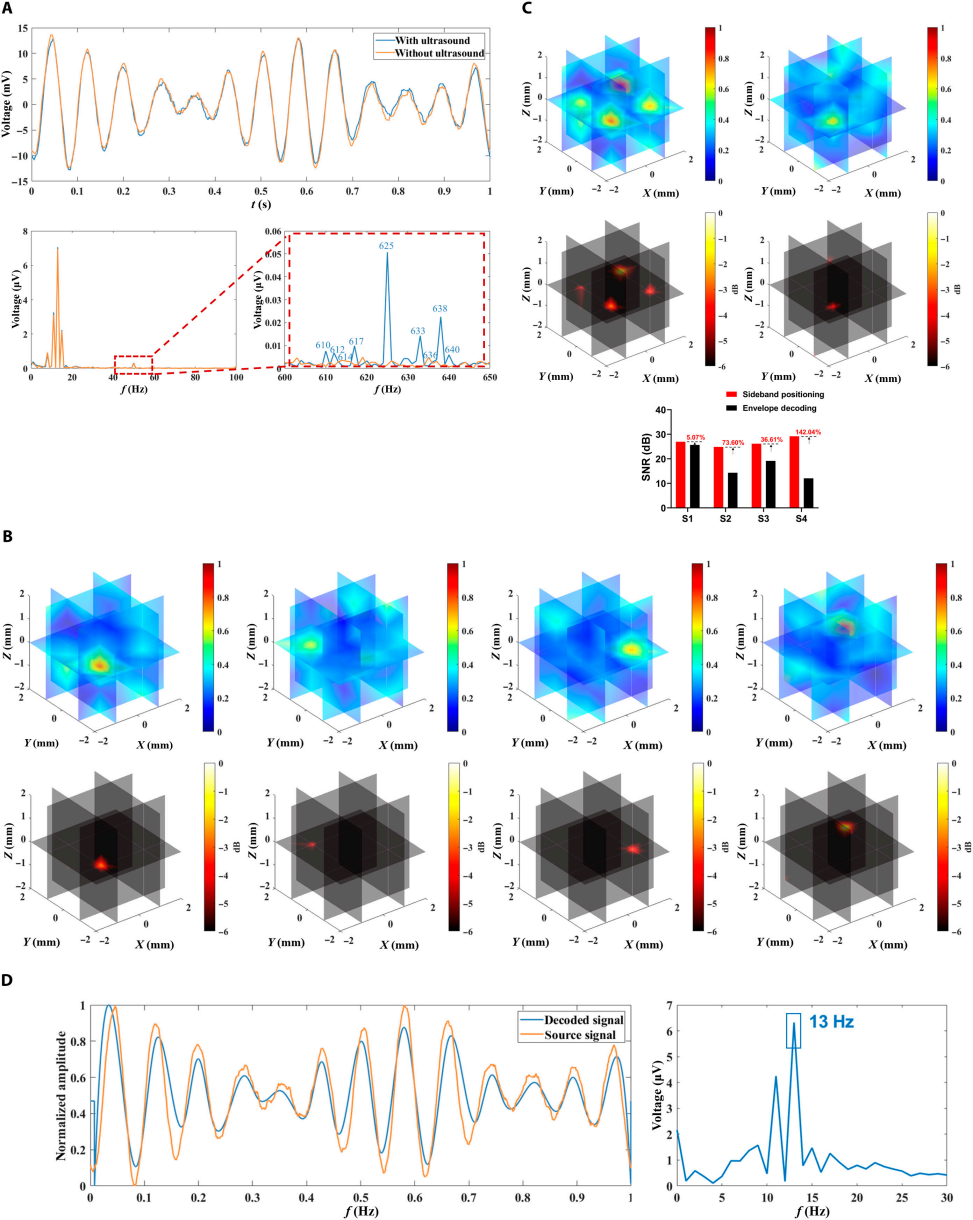
(A) Time–frequency plot of raw voltage signal with/without ultrasound irradiation. (B) PRF ± 8, PRF ± 11, PRF ± 13, and PRF ± 15 sideband frequency dipole localization results. (C) Comparison of PRF sideband localization and envelope decoding localization results. (D) Signal decoding plots for the multisource phantom experiment.

Four dipole source voltage signals with a discharge frequency of 8 Hz and a set position of (−1, −1, 0), a discharge frequency of 11 Hz and a set position of (−1, 1, 0), a discharge frequency of 13 Hz and a set position of (1, −1, 0), and a discharge frequency of 15 Hz and a set position of (1, 1, 0) were captured by Neuroscan as shown in Fig. 12A. From Fig. 12A, it can be seen that the voltage signal acquired without ultrasound irradiation does not have a band-specific signal component in the high-frequency band, and when ultrasonic irradiation is applied, under the influence of ultrasound modulation, the acquired voltage signal has a high-frequency component in a specific frequency band, and its specific frequency is the ultrasound’s PRF = 625 Hz, and there are also PRF ± 8, PRF ± 11, PRF ± 13, and PRF ± 15 of spectral peaks, i.e., 617 and 633, 614 and 636, 612 and 638, and 610 and 640 Hz for a total of 8 sideband spectral peaks. The USMEEG of the ultrasound scan raster points (5 × 5 × 5, totaling 125 scans) was processed by the PRF sideband localization algorithm. The dipole localization results and −6-dB attenuation thermograms obtained for the PRF ± 8, PRF ± 11, PRF ± 13, and PRF ± 15 sideband bands are shown in Fig. 12B. As shown in Fig. 12B, the sideband acoustoelectric value localization results and −6-dB decay heat maps for sideband frequencies of PRF ± 8, PRF ± 11, PRF ± 13, and PRF ± 15 have clear target points at the set position where the corresponding source signals are located at (−1, −1, 0), (−1, 1, 0), (1, −1, 0), and (1, 1, 0), respectively, whereas the sideband frequency localization in the other plots has no clear target point, indicating that the PRF sideband acoustoelectric values are able to accurately localize the dipole positions at the corresponding frequencies.

The PRF sideband localization results are obtained by averaging the superposition of the frequency values of each sideband and are compared with the dipole localization results obtained using the envelope decoding localization algorithm, the results of which are shown in Fig 12C. The specific values of the PRF sideband localization and envelope decoding localization for the 4 dipole sources localization SNRs are shown in Table 9. From the above results, it can be seen that the −6-dB decay heat map of PRF sideband localization has clear targets at the dipole source setting positions, and it is considered that PRF sideband localization can accurately locate 4 dipole sources, while the −6-dB decay heat map of envelope decoding localization has clear targets only at the S1 and S3 setting positions, and it is considered that envelope decoding localization can accurately locate only 2 dipole sources, and the SNR of the other dipole sources is too low. Comparing the localization SNRs of the 4 dipole sources, for S1, the envelope decoding localization is 25.64 dB and the PRF sideband localization is 26.94 dB, an improvement of 5.07%; for S2, the envelope decoding localization is 14.32 dB and the PRF sideband localization is 24.86 dB, an improvement of 73.60%; for S3, the envelope decoding localization is 19.15 dB and the PRF sideband is positioned at 26.16 dB, an improvement of 36.61%; and for S4, the envelope decoding is positioned at 12.06 dB and the PRF sideband is positioned at 29.19 dB, an improvement of 142.04%. Comparing the SNRs of the 4 dipole sources, the PRF sideband localization is markedly improved over the envelope decoding localization in all cases.

**Table 9.** Table of SNR values for dipole source localization

Source	PRF sideband positioning	Envelope decoding for localization
S1-SNR/dB	26.94	25.64
S2-SNR/dB	24.86	14.32
S3-SNR/dB	26.16	19.15
S4-SNR/dB	29.19	12.06

#### Decoding

The decoded signals obtained after processing the acquired multidipole source voltage signals by the decoding algorithm are shown in Fig. 12D. From the data in Fig. 12D, the Pearson correlation coefficient between the decoded signal and the source signal of the multisource body modeling experiment S3 is calculated to be 0.87.

## Discussion

This paper focuses on the numerical simulation and experimental study of USMEEG based on ultrasound phased array. A tFUS numerical simulation model and an experimental platform were established based on a real brain model and a 128-array phased array, and a 3D transcranial multisource dipole localization and decoding numerical simulation model and experimental platform were further constructed, and a high-precision localization and decoding algorithm was developed. Ultimately, the above research provides a theoretical basis and a technical solution for realizing the application of USMEEG to high-precision brain–computer control. However, in the work in this paper, the following points remain to be improved: Firstly, the focal size of the phased array in posttranscranial focusing is markedly larger than that in purely aqueous focusing, and further optimization of the focal size of the transcranially focused acoustic field by other optimization methods such as amplitude modulation will also be attempted subsequently. Furthermore, the short axis of the focal field of the transcranial state after the TR method is smaller than that of the pure water state, which may be due to the strong acoustic attenuation of the skull, resulting in a strong acoustic pressure attenuation still existing on both sides of the main focal point after the TR method, leading to the formation of the short axis of the focal field being narrower than that of the pure water state, and the other possible reason is that the shape of the focal field is related to the distance set by the skull and the surface of the transducer, and the distance between the skull and the surface of the transducer affects the transcranial focusing effect of ultrasound, and it is important to ensure that the 2 are at an appropriate distance to achieve an optimal transcranial focused acoustic field. Meanwhile, in order to carry out the subsequent USMEEG experiments smoothly, the acoustic bone material used in this work is a kind of ABS material with conductive properties, and its real electrical and acoustic properties are still different from the actual skull, and in the subsequent work, we will also try to use the skulls of real volunteers or develop a new kind of acoustic material based on the tissue-like phantom, which will provide more precise guidance for acoustic field measurements and USMEEG experiments. Secondly, the work in this paper only explores the imitation brain tissue phantom; compared to the complex brain, the experimental conditions are too ideal; the follow-up plan is to carry out USMEEG experiments based on isolated porcine brain and living animals.

The reasons why USMEEG is currently not widely used are considered in several ways. Firstly, how to make tFUS safely pass through the skull and accurately irradiate to the target area is the key to realizing USMEEG technology. Due to the heterogeneity of the skull and strong acoustic attenuation, the sound waves will gather in the skull during propagation, causing burns due to the high local energy in the skull, and the propagation path will also be changed, resulting in the inability of the ultrasound to accurately focus on the target area. Secondly, in order to avoid the problems of thermal damage to the scalp and brain tissue caused by too high an intensity of tFUS and the inability of too low an intensity to effectively modulate the EEG signal, the safe intensity threshold of tFUS and the effect of ultrasound on neuronal spontaneous discharges need to be further determined [33,34]. Finally, the current research on USMEEG is still in its infancy, mainly based on tissue mimicry and isolated physiological tissues, with the lack of animal and volunteer experiments, and extensive clinical trials are needed to verify its effectiveness and safety. In the work in this paper, the amplitude of the dipole source signal of the simulated discharge is large compared to the real EEG amplitude range, and the current acquired USMEEG is of microvolt level, and the amplitude magnitude of the USMEEG will be smaller for the real EEG magnitude, and the next plan is to optimize the acquisition circuitry for submicrovolt- or nanovolt-level USMEEG, in order to achieve improvement in the SNR of the acquired USMEEG and to realize microvolt- and nanovolt-level USMEEG acquisition. USMEEG acquisition provides effective technical means and methodological support for the application of USMEEG to noninvasive brain–computer and fine brain–computer control in general-purpose scenarios.

## Data Availability

The data used to support the findings of this study are included within the article.
